# Enabling Chemoenzymatic Strategies and Enzymes for Synthesizing Sialyl Glycans and Sialyl Glycoconjugates

**DOI:** 10.1021/acs.accounts.3c00614

**Published:** 2023-12-21

**Authors:** Xi Chen

**Affiliations:** Department of Chemistry, University of California, Davis, California 95616, United States

## Abstract

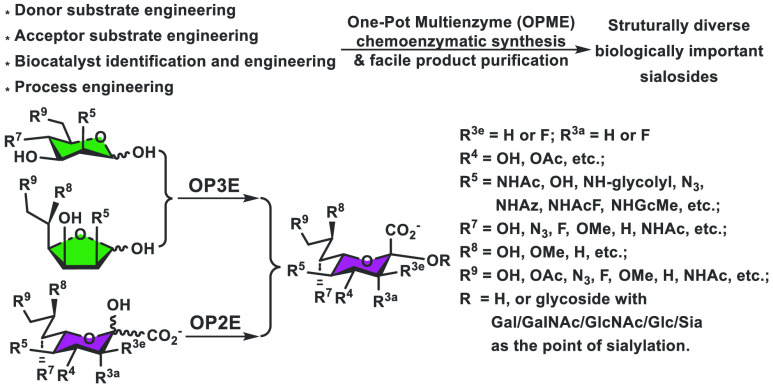

Sialic acids are fascinating negatively charged nine-carbon monosaccharides. Sialic acid-containing glycans and glycoconjugates are structurally diverse, functionally important, and synthetically challenging molecules. We have developed highly efficient chemoenzymatic strategies that combine the power of chemical synthesis and enzyme catalysis to make sialic acids, sialyl glycans, sialyl glycoconjugates, and their derivatives more accessible, enabling the efforts to explore their functions and applications. The Account starts with a brief description of the structural diversity and the functional importance of naturally occurring sialic acids and sialosides. The development of one-pot multienzyme (OPME) chemoenzymatic sialylation strategies is then introduced, highlighting its advantages in synthesizing structurally diverse sialosides with a sialyltransferase donor substrate engineering tactic. With the strategy, systematic access to sialosides containing different sialic acid forms with modifications at C3/4/5/7/8/9, various internal glycans, and diverse sialyl linkages is now possible. Also briefly described is the combination of the OPME sialylation strategy with bacterial sialidases for synthesizing sialidase inhibitors. With the goal of simplifying the product purification process for enzymatic glycosylation reactions, glycosphingolipids that contain a naturally existing hydrophobic tag are attractive targets for chemoenzymatic total synthesis. A user-friendly highly efficient chemoenzymatic strategy is developed which involves three main processes, including chemical synthesis of lactosyl sphingosine as a water-soluble hydrophobic tag-containing intermediate, OPME enzymatic extension of its glycan component with a single C18-cartridge purification of the product, followed by a facile chemical acylation reaction. The strategy allows the introduction of different sialic acid forms and diverse fatty acyl chains into the products. Gram-scale synthesis has been demonstrated. OPME sialylation has also been demonstrated for the chemoenzymatic synthesis of sialyl glycopeptides and *in vitro* enzymatic N*-*glycan processing for the formation of glycoproteins with disialylated biantennary complex-type N-glycans. For synthesizing human milk oligosaccharides (HMOs) which are glycans with a free reducing end, acceptor substrate engineering and process engineering strategies are developed, which involve the design of a hydrophobic tag that can be easily installed into the acceptor substrate to allow facile purification of the product from enzymatic reactions and can be conveniently removed in the final step to produce target molecules. The process engineering involves heat-inactivation of enzymes in the intermediate steps in multistep OPME reactions for the production of long-chain sialoside targets in a single reaction pot and with a single C18-cartridge purification process. In addition, a chemoenzymatic synthon strategy has been developed. It involves the design of a derivative of the sialyltransferase donor substrate precursor, which is tolerated by enzymes in OPME reactions, introduced to enzymatic products, and then chemically converted to the desired target structures in the final step. The chemoenzymatic synthon approach has been used together with the acceptor substrate engineering method in the synthesis of complex bacterial glycans containing sialic acids, legionaminic acids, and derivatives. The biocatalysts characterized and their engineered mutants developed by the Chen group are described, with highlights on synthetically useful enzymes. We anticipate further development of chemoenzymatic strategies and biocatalysts to enable exploration of the sialic acid space.

## Key References

- YuH.; ChokhawalaH.; KarpelR.; YuH.; WuB.; ZhangJ.; ZhangY.; JiaQ.; ChenX.A multifunctional *Pasteurella multocida* sialyltransferase: a powerful tool for the synthesis of sialoside libraries. J. Am. Chem. Soc.2005, 127, 17618–1761910.1021/ja056169016351087 .^1^ An early and representative example of sialyltransferase donor substrate engineering for the chemoenzymatic synthesis of sialosides containing diverse sialic acid forms and derivatives using a one-pot multienzyme (OPME) sialylation chemoenzymatic strategy with substrate promiscuous enzymes from bacterial sources.
- SugiartoG.; LauK.; QuJ.; LiY.; LimS.; MuS.; AmesJ. B.; FisherA. J.; ChenX.A sialyltransferase mutant with decreased donor hydrolysis and reduced sialidase activities for directly sialylating LewisX. ACS Chem. Biol.2012, 7, 1232–124010.1021/cb300125k22583967 PMC3521065.^2^ Crystal structure-based biocatalyst engineering for the generation of a synthetically useful sialyltransferase mutant that can directly sialylate fucosylated acceptor substrates for the highly efficient synthesis of sialyl Lewis x and sialyl Lewis a structures.
- BaiY.; YangX.; YuH.; ChenX.Substrate and process engineering for biocatalytic synthesis and facile purification of human milk oligosaccharides. ChemSusChem2022, 15, e20210253910.1002/cssc.20210253935100486 PMC9272545.^3^ An example of acceptor substrate and process engineering strategies for the facile chemoenzymatic synthesis of glycans with a single purification process to access even highly complex glycan targets.
- YuH.; ZhangL.; YangX.; BaiY.; ChenX.Process engineering and glycosyltransferase improvement for short route chemoenzymatic total synthesis of GM1 gangliosides. Chem.—Eur. J.2023, 29, e20230000510.1002/chem.20230000536596720 PMC10159885.^4^ A user-friendly highly efficient chemoenzymatic strategy showcased for the synthesis of complex gangliosides on gram scales by the one-pot enzymatic glycosylation of chemically synthesized lactosyl sphingosine followed by chemical acylation, with highlights of short-route chemical synthesis, glycosylation process engineering, and biocatalyst improvement.

## Introduction

**Figure 1:**
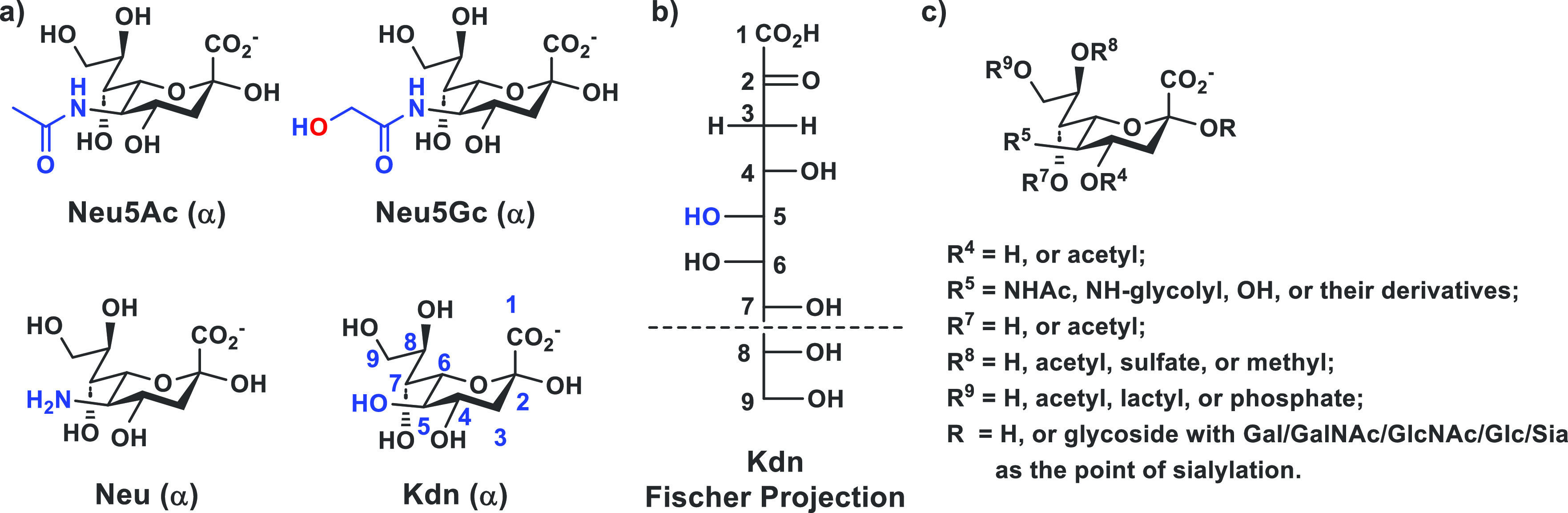
a) Structures (α-anomers are shown) of the most abundant sialic acid form *N*-acetylneuraminic acid (Neu5Ac), nonhuman animal *N*-glycolylneuraminic acid (Neu5Gc), their parent monosaccharide neuraminic acid (Neu), and 2-keto-3-deoxy-d-glycero-d-galacto-nononic acid or 2-keto-3-deoxy-nononic acid (Kdn); b) the Fischer projection of Kdn; and c) diverse naturally occurring sialic acid forms.

Sialic acids (Figure 1), subset members of the α-keto acid-type nine-carbon monosaccharide family called nonulosonic acids (NulOs),^5^ are remarkably fascinating molecules on several fronts from both chemistry and biology points of views. They are well known for their structural complexity and diversity, chemical synthetic challenges, biological importance, and potential for therapeutic development.

**Figure 2:**
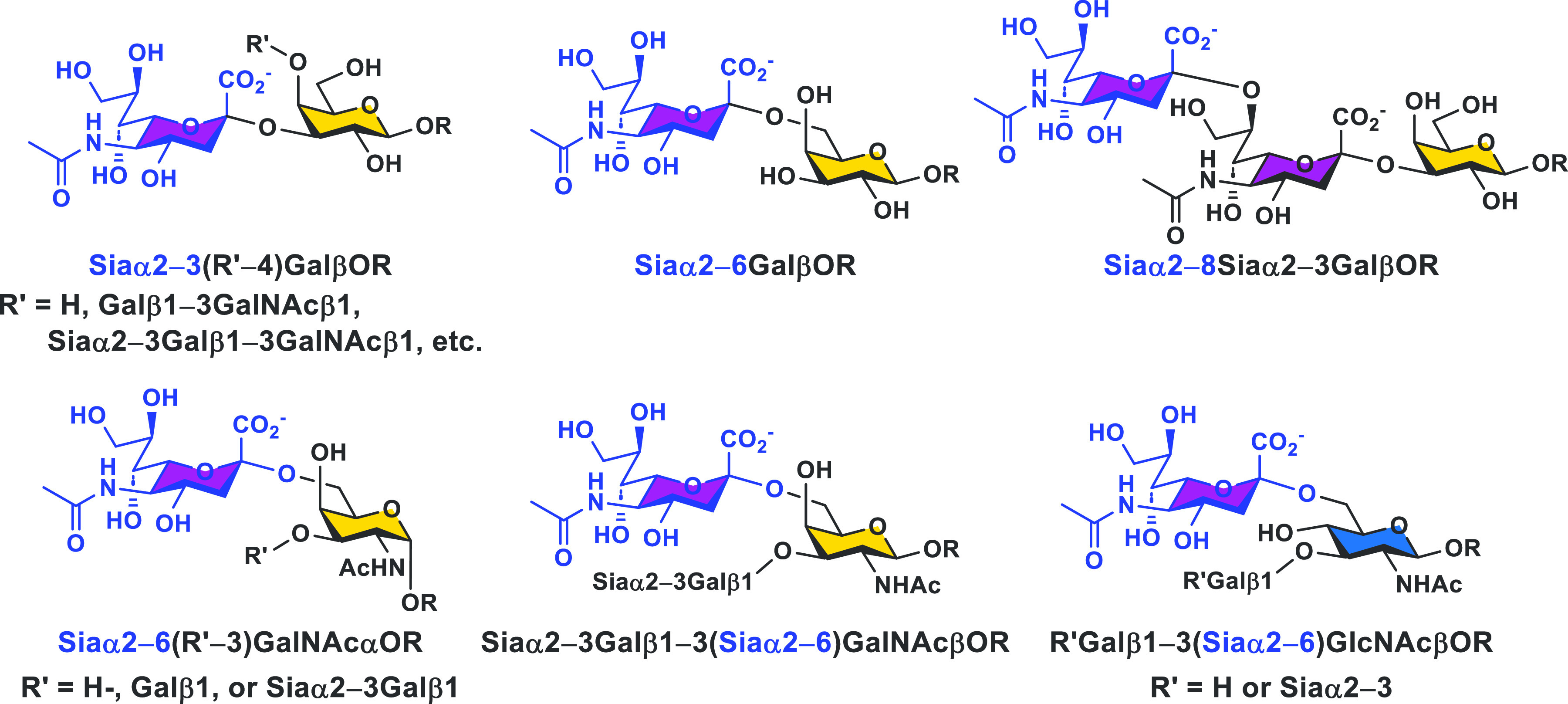
Common sialyl linkages and structural components found in mammalian glycans and glycoconjugates.

The most abundant sialic acid form in nature is *N*-acetylneuraminic acid (Neu5Ac), which is a derivative of neuraminic acid (Neu) with an *N*-acetyl group substitution at its carbon-5 amino group (Figure 1a). Another *N*-acyl derivative of Neu, *N*-glycolylneuraminic acid (Neu5Gc) with an extra oxygen atom at its C5-*N*-acyl group compared to Neu5Ac, is abundant in animals but is not biosynthesized by humans. On the other hand, 2-keto-3-deoxy-d-glycero-d-galacto-nononic acid (Figure 1b) or 2-keto-3-deoxy-nononic acid (Kdn) that is more common in cold-blooded vertebrates^6^ differs from Neu by replacing its C5-amino group with a hydroxyl group. Further modification of Neu5Ac, Neu5Gc, and Kdn at a single site or at multiple sites at C4, C5, C7, C8, and/or C9 (Figure 1c) can occur in nature, with more than 50 different sialic acid forms having been identified and *O*-acetylation as the most common sialic acid modification.^5,7,8^

Sialic acids are common terminal monosaccharides that are α2–3 or α2–6-linked to a galactose (Gal), α2–8-linked to another sialic acid (Sia), or α2–6-linked to an *N*-acetylgalactosamine (GalNAc) or an *N*-acetylglucosamine (GlcNAc) (Figure 2) in mammalian sialyl glycans or glycoconjugates and in surface carbohydrates produced by some pathogenic bacteria. Sialic acids with additional sialyl linkages and in different contexts have also been found in the capsular polysaccharides (CPSs), the lipopolysaccharides, and the cell walls of other bacteria.^5,7,9^

In mammals, sialic acids are involved in many molecular interactions that influence numerous biological processes, including cell–cell interaction, signal transduction, inflammation, immune regulation, cancer metastasis, bacterial and viral infection, and others. They are important for brain development and also influence the stability, targeting, and serum half-life of glycoprotein therapeutics.^5,7,9^ Nevertheless, the details of the structure–function relationship of many of these events are lacking, mainly due to the limited access to efficient tools and structurally defined compounds.

We have been interested in designing and developing enabling chemoenzymatic synthetic strategies that combine the power of chemical synthesis and enzyme catalysis to obtain structurally defined sialic acid-containing glycans and glycoconjugates with facile purification processes. We are also interested in exploring their functions in our own laboratory and with our collaborators. This Account focuses on the enzymes involved and the key chemoenzymatic strategies developed for sialoside synthesis, including one-pot multienzyme (OPME) sialylation systems and strategies of sialyltransferase donor and acceptor substrate engineering to introduce different sialic acid forms and allow facile product purification, process engineering to streamline product formation and minimize purification procedures, and biocatalyst identification and engineering. These systems and strategies are presented with examples for synthesizing glycan probes, sialidase inhibitors, glycosphingolipids, glycopeptides, glycoproteins, human milk oligosaccharides (HMOs), and bacterial glycans.

## One-Pot Multienzyme (OPME) Chemoeznymatic Synthesis of Sialyl Glycans Containing Different Sialic Acid Forms: Sialyltransferase Donor Substrate Engineering

Sialyltransferases are nature’s key enzymes for the formation of sialyl linkages. As sialic acid-containing biomolecules are well-known challenging targets for chemical synthesis, enzymatic syntheses, especially those using sialyltransferases, are particularly useful. Pioneering efforts by James Paulson, Chi-Huey Wong, and others in the late 1980s and early 1990s used purified mammalian sialyltransferases with or without *in situ* sugar nucleotide regeneration for synthesizing sialoglycans. The identification and cloning of bacterial sialyltransferases by the groups of Warren Wakarchuk, Chi-Huey Wong, and Takeshi Yamamoto et al. at Japan Tobacco Inc. in the late 1990s and early 2000s expanded the applications of sialyltransferases in synthesis. The focus was on targets containing Neu5Ac, although cytidine 5′-monophosphate-sialic acids (CMP-Sias) containing Neu5Gc, 9-*O*-acetyl Neu5Ac (Neu5,9Ac_2_), or other C-9-modified Neu5Ac were reported and used for testing the donor substrate specificities of mammalian sialyltransferases. When we began our exploration of the sialic acid space in 2003, many different sialic acid forms had been discovered from nature, but the related sialosides were mostly synthetically unavailable. My then new group aimed to develop chemoenzymatic methods to access sialyl glycans containing different sialic acid forms, including those found in nature and those containing non-natural modifications.

**Figure 3:**
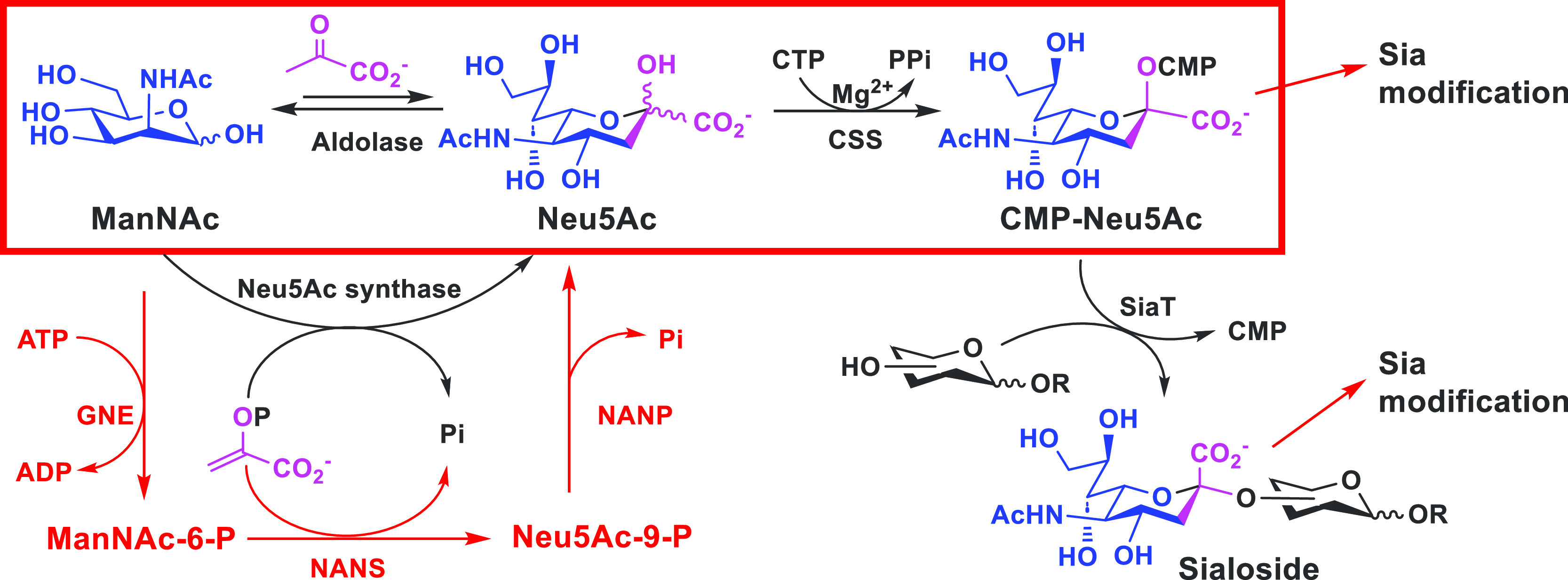
Nature’s sialoside biosynthetic routes.^9^ In eukaryotes, *N*-acetylmannosamine (ManNAc) and adenosine 5′-triphosphate (ATP) are used by the ManNAc 6-phosphokinase activity of a bifunctional enzyme GNE to form ManNAc-6-phosphate (ManNAc-6-P), which is used together with phosphoenolpyruvate (PEP) by Neu5Ac-9-phosphatate synthase (NANS) to form Neu5Ac-9-P that is dephosphorylated by Neu5Ac-9-P phosphatase (NANP) to form Neu5Ac. In bacteria, ManNAc is used directly by Neu5Ac synthase in the presence of PEP to form Neu5Ac. Sialic acid aldolase catalyzing the reversible reaction of sialic acid degradation has been used commonly to prepare Neu5Ac from ManNAc and pyruvate. Neu5Ac is activated by a CMP-sialic acid synthetase (CSS) in the presence of cytidine 5′-triphosphate (CTP) to form CMP-Neu5Ac, the donor substrate of sialyltransferases for producing sialosides. Naturally occurring sialic acid modifications are introduced at the CMP-Neu5Ac or the sialoside stage by enzyme-catalyzed reactions.^9^

Our initial targets were sialosides representing the common terminal glycan components of mammalian cell surface glycoproteins and glycolipids. Sialyltransferases determine the structures of the products formed, including sialic acid forms, sialyl linkages, and underlying glycans, and they use CMP-activated sialic acids as donor substrates (Figure 3). CMP-sialic acids are formed from sialic acids and cytidine 5′-triphosphate (CTP) by CMP-sialic acid synthetases in both eukaryotes and bacteria. The formation of sialic acids differs in these systems. Some pathogenic bacteria do not have their own complete *de novo* sialoside biosynthetic pathway^9^ and scavenge either CMP-sialic acids or free sialic acids from hosts. Some bacteria form Neu5Ac from a six-carbon monosaccharide, *N*-acetylmannosamine (ManNAc), and phosphoenolpyruvate (PEP) by Neu5Ac synthase; and in eukaryotic systems, ManNAc is converted to ManNAc-6-phosphate that reacts with PEP to form Neu5Ac-9-phosphate, which is then used to form Neu5Ac, with the involvement of three enzymes (Figure 3). Nevertheless, Neu5Ac in both systems can be degraded to ManNAc and pyruvate by sialic acid aldolases (or *N*-acetylneuraminate lyases), which catalyze reversible reactions, and the reverse reaction has been used broadly for the enzymatic synthesis of sialic acids from their six-carbon precursors and pyruvate.

**Figure 4:**
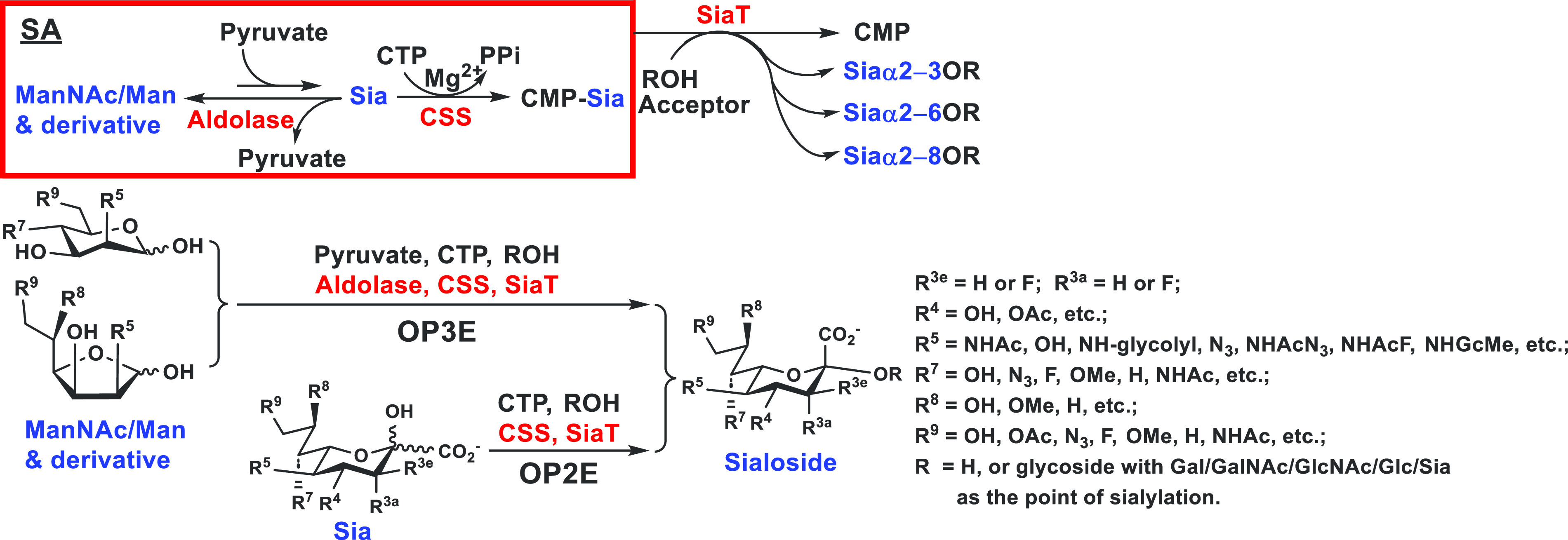
One-pot multienzyme (OPME) sialylation systems for synthesizing α2–3-, α2–6-, and α2–8-linked sialosides containing different sialic acid forms and derivatives with a one-pot three-enzyme (OP3E) or a one-pot two-enzyme (OP2E) system.

While naturally occurring sialic acid modifications are introduced at the CMP-Neu5Ac or the sialoside stage by enzyme-catalyzed reactions (Figure 3),^9^ we envisioned that it would be possible to use three enzymes including a sialic acid aldolase, a CMP-sialic acid synthetase, and a sialyltransferase in one pot to produce sialosides containing different sialic acid forms from chemically modified six-carbon precursors of sialic acids or derivatives. Indeed, by using the combination of a CMP-sialic acid synthetase (CSS) and sialyltransferases with or without a sialic acid aldolase in a one-pot three-enzyme (OP3E) or a one-pot two-enzyme (OP2E) sialylation system,^10^ sialosides containing a variety of sialic acid forms, different sialyl linkages, and diverse underlying glycans have been synthesized (Figure 4). For example, α2–3- and α2–6-linked sialosides containing a sialic acid with a substitution at C3, C4, C5, C7, C8, and/or C9 have been obtained.^1,10−17^ Disialyl glycans containing a structurally varied terminal α2–8-linked sialic acid^18,19^ have also been produced. These sialosides have been used to generate glycoconjugates, to print glycan microarrays, and as probes by our collaborators for functional studies of sialic acid-binding proteins and antibodies. The underlying glycans in sialosides were initially chemically synthesized for enzymatic sialylation but were later formed chemoenzymatically using OPME systems that we developed. All OPME systems start with a simple monosaccharide or derivative, which is activated to form a sugar nucleotide as the donor substrate for the glycosyltransferase in the system to produce a target carbohydrate with the desired glycosidic linkage.

Quite remarkably, *E. coli* sialic acid aldolase was shown to tolerate even disaccharides containing a mannose derivative at the free reducing end as substrates for synthesizing disaccharides containing a free sialic acid at the reducing end.^20^*Photobacterium damselae* α2–6-sialyltransferase (Pd2,6ST) was shown to catalyze the block transfer of oligosaccharide analogs from the corresponding CMP donors to produce size-defined polysaccharide analogs^21^ and a novel class of macrocyclic oligosaccharides.^22^

OPME sialylation systems were applied for the construction of a comprehensive library of structurally diverse α2–3- and/or α2–6-linked sialyl Galβ*p*NP structures containing a sialic acid with the hydroxyl group at C-5,^14^ C-9,^17^ or C-7^15^ systematically substituted with a hydrogen, a fluorine, an azido, or a methoxy group. In addition, sialyl Galβ*p*NP compounds containing 9-*O*-acetyl Neu5Ac (Neu5,9Ac_2_)^23^ and its stable 9-*N*-acetyl Neu5Ac analog (Neu5Ac9NAc),^24^ 4-*O*-acetyl Neu5Ac (Neu4,5Ac_2_) and 4-*O*-acetyl Neu5Gc (Neu5Gc4Ac),^13^ 7-*N*-acetyl Neu5Ac (Neu5Ac7NAc)^25^ and a related 9-deoxy bacterial nonulosonic acid 5,7-di-*N*-acetyllegionaminic acid (Leg5,7diNAc),^26^ and 7,9-di-*N*-acetyl Neu5Ac (Neu5Ac7,9diNAc)^25^ have been synthesized. Disialyl Galβ*p*NP compounds containing a structurally varied terminal α2–8-linked sialic acid have also been produced.^19^ These compounds have been used in coupled enzymatic assays for high-throughput substrate specificity studies^23^ of sialidases from human, bacteria, and human and avian influenza A viruses. In these assays, an excess amount of a β-galactosidase was included in the reaction mixture to release *p*NP (which was quantified calorimetrically) from Galβ*p*NP formed by the sialidase-catalyzed cleavage of sialyl Galβ*p*NP.^23^

**Figure 5:**
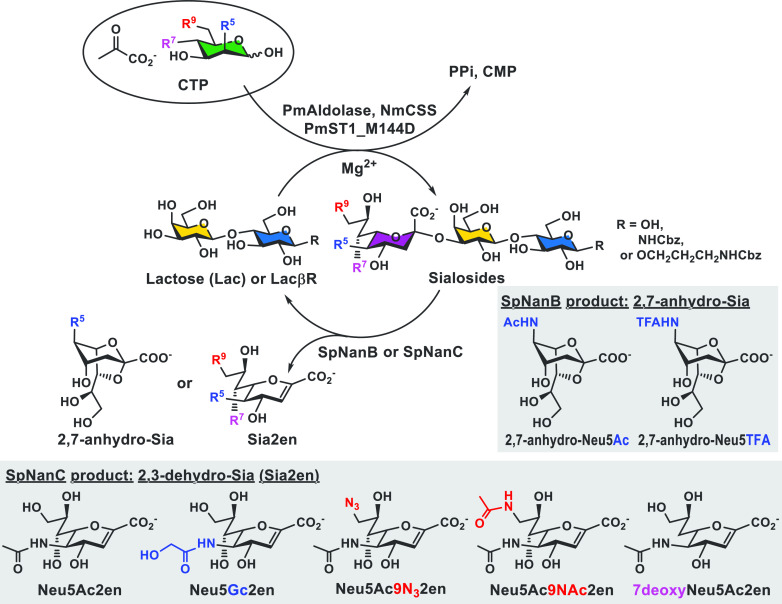
One-pot multienzyme (OPME) synthesis of sialidase inhibitors 2,7-anhydro-Sias or Sia2ens by combining an OPME sialylation system with bacterial sialidase SpNanB or SpNanC, respectively. Adapted with permission from ref (10). Copyright 2019 Elsevier. Data from refs (27−29.)

Information learned from sialidase substrate specificity studies was used to design inhibitors selectively against specific sialidases. Bacterial sialidases SpNanB and SpNanC from *Streptococcus pneumoniae* have also been included in the OPME chemoenzymatic synthetic scheme to form useful sialidase inhibitors and probes including 2,7-anhydro-sialic acids (2,7-anhydro-Sias) and 2,3-dehydro sialic acids (Sia2ens), respectively (Figure 5).^27,28^ The method was further improved using a benzyl carbamate (NHCbz)-tagged sialyltransferase acceptor which can be recycled for the gram-scale production of 2,7-anhydro-Neu5Ac by SpNanB.^29^

The combination of 2,3-dehydro-*N*-glycolylneuraminic acid (Neu5Gc2en) and 2,3-dehydro-*N*-acetylneuraminic acid (Neu5Ac2en) (Figure 5) was shown to protect mice against sepsis^30^ and against endotoxic shock^31^ by our collaborator Yang Liu’s group. The azido-containing Sia2en was further derivatized using click reactions to form inhibitors selective against certain bacterial sialidases.^32^ 2,3-Difluoro-Neu5Ac (DFNeu5Ac) and the related 9-N_3_ derivatives (DFNeu5Ac9N_3_) were also chemoenzymatically synthesized using a PmAldolase- or EcAldolase-catalyzed reaction, and (2-*equatorial*-3-*axial*)-difluoro-9-azido-Neu5Ac (2*e*3*a*DFNeu5Ac9N_3_) was shown to be a long-lasting nanomolar mechanism-based inhibitor selectively against sialidases from bacterial pathogens *Clostridium perfringens* and *Vibrio cholerae*.^33^ 2,7-Anhydro-Neu5Ac was shown by our collaborator Nathalie Juge’s group to be a suitable sole carbon source and a potential prebiotic for a human gut commensal bacterium *Ruminoccocus gnavus*.^34^

We soon realized that once suitable enzymes were identified and obtained, the synthesis of target compounds using OPME glycosylation systems was straightforward, but the product purification was not easy. Multiple chromatography processes might be needed to obtain pure products. Purification processes for glycan products with a free reducing end were particularly challenging and could require the use of a semipreparation high-performance liquid chromatography system such as the case for the OPME synthesis of glycosphingolipid glycans.^35^

To simplify product purification, we explored the strategy of attaching a hyperfluorous tag to glycosyltransferase acceptors,^36^ inspired by the work of Nicola Pohl’s group in using fluorous-tagged glycans for chemical synthesis and for glycan microarray studies. We found that although the strategy simplified the product purification, the necessity of adding a suitable linker to make the acceptors compatible with glycosyltransferase-catalyzed reactions^36^ and the multistep synthetic process to obtain the suitable fluorous-tagged acceptors were time-consuming and required expensive reagents which were not desirable for large-scale synthesis. Furthermore, the tag was not readily removable from the product, leading to products with an undesired unnatural aglycone component.

## Chemoenzymatic Total Synthesis of Glycosphingolipids (GSLs)

Encouraged by streamlining the product purification process of enzymatic glycosylation reactions using fluorous-tagged glycosyltransferase acceptors, we identified glycosphingolipids (GSLs) as attractive targets for our OPME chemoenzymatic synthetic strategies. Sialic acid-containing GSLs called gangliosides are present on all mammalian plasma membranes and are particularly abundant in the nervous systems. They play important roles in lipid raft formation, cell–cell recognition, signal transduction regulation, neuronal plasticity, inflammation, immune regulation, cancer progression, bacterial and viral infections, etc. Some gangliosides are being developed as cancer markers, cancer vaccine candidates, immune suppressants, and therapeutics for treating neural damage and neural diseases including Huntington’s and Parkinson’s diseases.^37^ Structurally defined synthetic GSLs are important standards and probes for research, but those from commercial sources are limited and expensive, and the GSLs isolated from nature are mixtures with the same glycan but different fatty acyl chains and/or sphingosines. We envisioned that a user-friendly chemoenzymatic method could be developed to allow even nonspecialists to produce structurally defined complex GSLs in their own laboratories.

**Figure 6:**
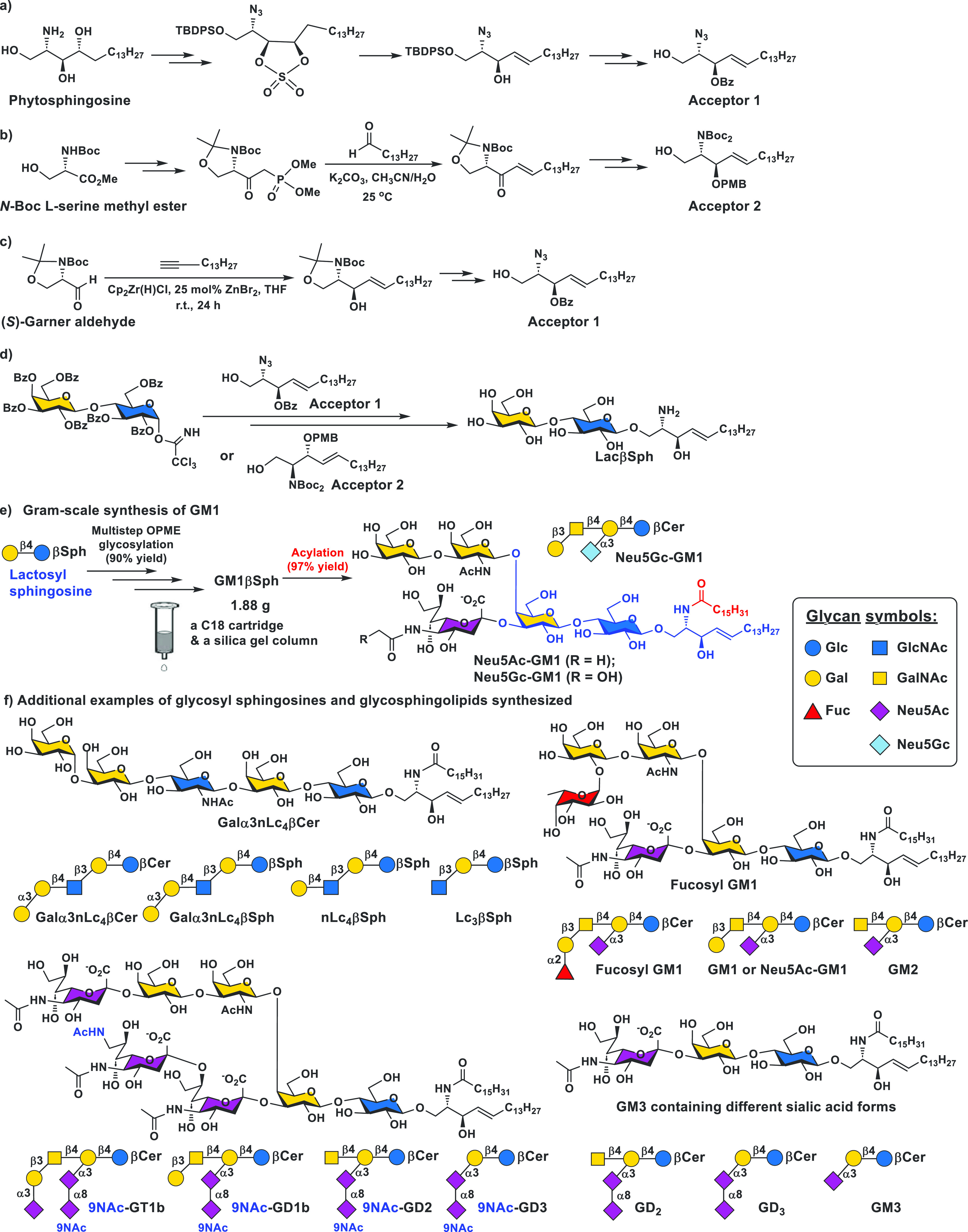
Three chemical synthetic approaches for the formation of sphingosine acceptors from (a) phytosphingosine, (b) *N*-Boc l-serine methyl ester and tetradecanoyl aldehyde, or (c) (*S*)-Garner’s aldehyde and 1-pentadecyne for (d) synthesizing LacβSph by the chemical glycosylation of a per-*O*-benzoylated trichloroacetimidate lactosyl donor. (e) An example of the gram-scale synthesis of GM1βSph from LacβSph in a multistep OPME enzymatic glycosylation process followed by chemical acylation for the formation of GM1 containing either Neu5Ac or Neu5Gc. (f) Structures and symbol representations of other glycosyl sphingosines and glycosphingolipids that have been synthesized.

All complex vertebrate GSLs share a common lactosyl ceramide (LacβCer) core that is not soluble in water and not a suitable acceptor substrate for the *in vitro* enzymatic synthesis of more complex GSLs in aqueous solution. On the other hand, lactosyl sphingosine (LacβSph) lacking the fatty acyl chain in LacβCer is readily soluble in water^38^ and is an ideal intermediate for OPME glycosylation to allow the facile purification of products using a simple C18 cartridge. To access LacβSph as an important intermediate, we developed several chemical synthetic methods with major variations in the preparation of sphingosine acceptors for chemical glycosylation. The first approach started from phytosphingosine^38,39^ via a 1-*O*-*tert*-butyldiphenylsilyl (TBDPS)-protected 2-azido derivative of a 3,4-cyclic sulfate intermediate for the formation of the 4-*E*-allylic alcohol structural feature in the azido- derivative of the sphingosine acceptor (Acceptor 1, Figure 6a). Its application was demonstrated for a 13-*g*-scale synthesis of LacβSph.^39^ Nevertheless, phytosphingosine is relatively expensive and does not allow for easy variation of the sphingosine chain length. The second approach developed by our collaborators Peng G. Wang and Jun Yin’s groups started from an inexpensive *N*-Boc l-serine methyl ester to form a β-ketophosphonate intermediate which reacted with an alkyl aldehyde in the presence of potassium carbonate in acetonitrile and water to form exclusively the desired *E*-olefin derivative in the sphingosine acceptor (Acceptor 2, Figure 6b) via the Horner–Wadsworth–Emmons reaction.^40^ The third approach used (*S*)-Garner’s aldehyde and 1-pentadecyne as starting materials to form the sphingosine Acceptor 1 (Figure 6c) in a short four-step route with two column purification steps.^4^ Chemical glycosylation of Acceptor 1 or Acceptor 2 with a per-*O*-benzoylated trichloroacetimidate lactosyl donor followed by deprotection formed the desired LacβSph (Figure 6d) ready for enzymatic glycosylation.

Complex glycosyl sphingosines, including those in the neolacto series (e.g., Lc_3_βSph, nLc_4_βSph, and Galα3nLc_4_βSph)^38^ and the ganglio series, were readily produced from LacβSph using sequential OPME reactions. The ganglio-series GSLs obtained included prioritized cancer antigens GM3, fucosyl GM1, GD3, and GD2;^39^ GM1 (Figure 6e), which is a therapeutic candidate for Huntington’s and Pakinson’s diseases;^4^ and 9-*N*-acetyl analogs of 9-*O*-acetylated b-series ganglio-series gangliosides including 9NAc-GD3, 9NAc-GD2, 9NAc-GD1b, and 9NAc-GT1b (Figure 6f).^41^ The sphingosine hydrophobic tail in the glycosyl sphingosine product facilitated the product purification from OPME reactions in less than 30 min with a single C18 cartridge, which was much simpler compared to the multiple column purification processes needed for purifying glycosphingolipid glycans containing a free reducing end^35^ or a propyl azide aglycone.^18,42^ Initial synthesis involved purifying individual intermediate glycosyl sphingosines, which was necessary when they were all desired targets^39^ but was not needed when a more complex GSL such as GM1 was the target.^4^ The use of the OPME sialylation system allowed the introduction of different sialic acid structures to the products as demonstrated for the synthesis of GM3 glycosphingosines containing various sialic acid forms.^40^ The fatty acyl chain was added to the glycosyl sphingosine product in the last step to form target GSLs^38^ with the advantage of the possibility of introducing different fatty acyl structures as demonstrated for GM3 synthesis,^40^ showcasing the flexibility of the chemoenzymatic total synthetic method. The acylation conditions were improved from a 24 h reaction^38^ by reacting with a fatty acid to a 2 h reaction by reacting with a fatty acyl chloride in a mixed solvent of tetrahydrofuran (THF) and saturated sodium bicarbonate (NaHCO_3_) aqueous solution (1:1, by volume).^39^ The efficiency of the acylation reaction was further improved by changing the mixed solvent by replacing the saturated NaHCO_3_ to 1% sodium carbonate (Na_2_CO_3_).^41^

## Glycopeptides and Glycoproteins

Glycopeptides are another family of glycoconjugates with a hydrophobic tail that facilitates C18 cartridge purification of the product from enzymatic reactions. A GalNAc-MUC1 glycopeptide was produced by solid-phase chemical synthesis and used for enzymatic glycosylation to form a T-MUC1 glycopeptide, as well as STn-MUC1 and ST-MUC1 glycopeptides containing Neu5Ac, Neu5Gc, or a derivative with an azido group at C9 or C5.^43^

*In vitro* processing of *N*-glycans on glycoproteins from high-mannose types to α2–3- or α2–6-disialylated biantennary complex types was achieved using recombinant glycosyltransferases expressed in *Escherichia coli*, including those from human and bovine origins.^44^ In this case, recombinant enzymes, all with a His_6_ tag, could be readily removed by absorption with nickel nitrilotriacetic acid resin, and the relatively large size of glycoprotein products helped their purification by dialysis or ultrafiltration to remove small molecular reagents.

## Chemoenzymatic Synthesis of Human Milk Oligosaccharides (HMOs) with Acceptor Substrate Engineering and Process Engineering Strategies

As described above, the naturally existing sphingosine in glycosyl sphingosines and the peptide in glycopeptides worked well as hydrophobic tags for the facile purification of products from OPME reaction mixtures. For human milk oligosaccharides (HMOs)^45^ that do not have a hydrophobic tail, we envisioned that engineering acceptor substrates with an easy-to-install and easy-to-remove hydrophobic tag would facilitate the product purification process.

Human milk oligosaccharides (HMOs) are attractive synthetic targets due to their prebiotic, antimicrobial, immunomodulation, and brain development nutritional potentials.^3,45^ Our early efforts to synthesize HMOs and analogs as well as Neu4,5Ac_2_-containing monotreme milk oligosaccharides^13^ started directly from lactose or other oligosaccharides with a free reducing end using OPME or sequential OPME strategies which involved labor-intense purification processes and the use of relatively large amounts of solvents.

**Figure 7:**
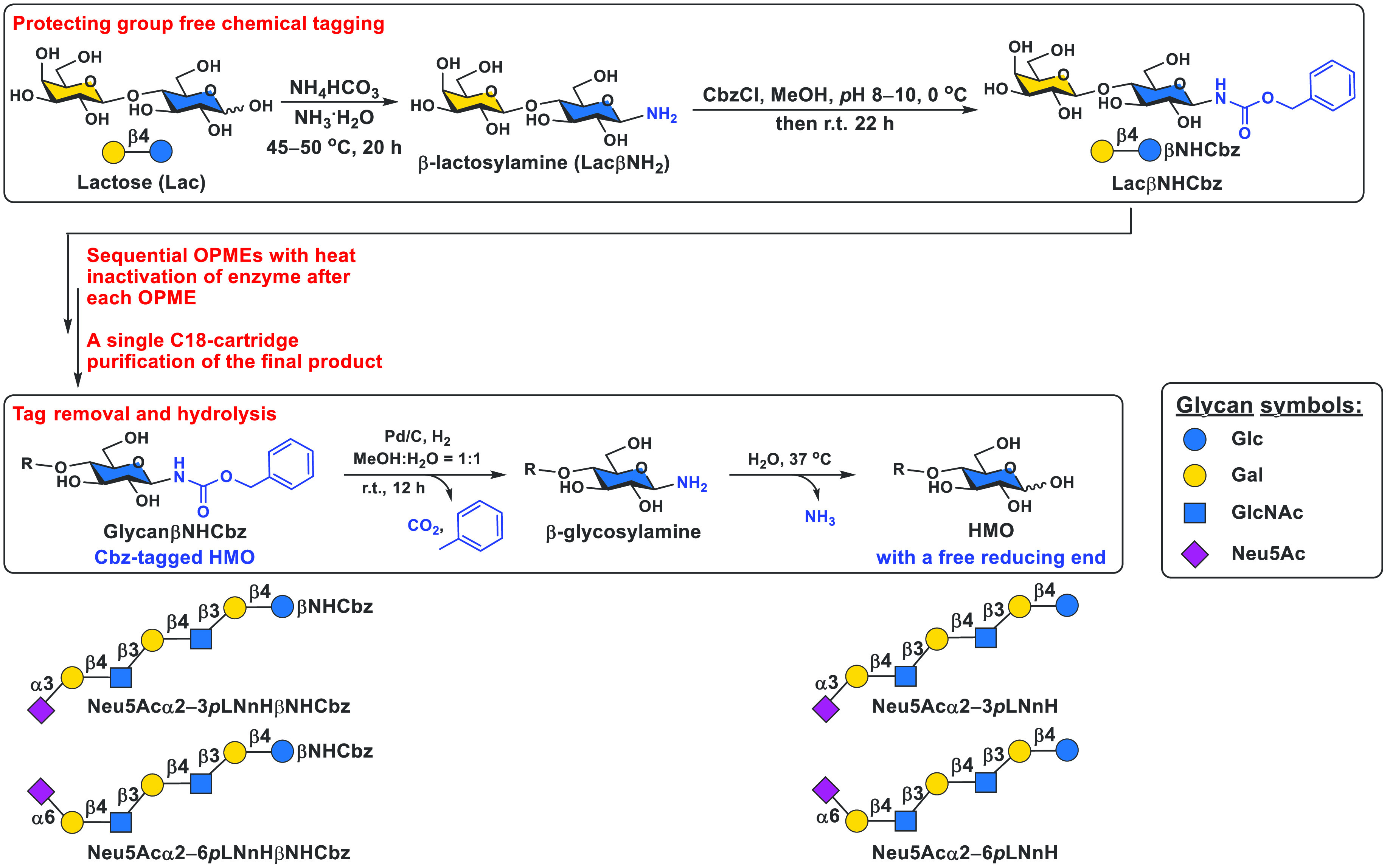
Acceptor substrate engineering and process engineering strategies for the multistep OPME chemoenzymatic synthesis of human milk oligosaccharides (HMOs) including sialylated ones with Neu5Acα2–3*p*LNnH and Neu5Acα2–6*p*LNnH shown as examples.

To simplify the enzymatic synthesis and purification of HMOs, we designed carboxybenzyl (Cbz)-tagged lactosylamine (LacβNHCbz)^3,29^ as an important intermediate. It was easily produced from inexpensive lactose in a two-step protection-group-free reaction (Figure 7) and was a superb substrate for glycan extension by glycosyltransferase-based OPME reactions to form a diverse array of NHCbz-tagged HMOs. Instead of purifying the intermediate product after every OPME reaction step, we found that deactivating enzymes in the OPME reaction before adding enzymes for the next OPME reaction was highly effective in precisely controlling the product structure and length to produce the desired target, despite its length, in one pot without the need to purify intermediate glycans. The combined acceptor substrate engineering and process engineering strategies with the multistep OPME process have been demonstrated for the high-yield production of more than 20 NHCbz-tagged HMOs (up to nonasaccharides) from LacβNHCbz with a single C18 cartridge-purification process. The NHCbz tag was readily removed from the products by catalytic hydrogenation followed by hydrolysis to form desired naturally existing HMOs with a free reducing end without the need for column purification.^3^

## Chemoenzymatic Synthesis of Bacterial Glycans with Acceptor Substrate Engineering and Chemoenzymatic Synthon Strategies

**Figure 8:**
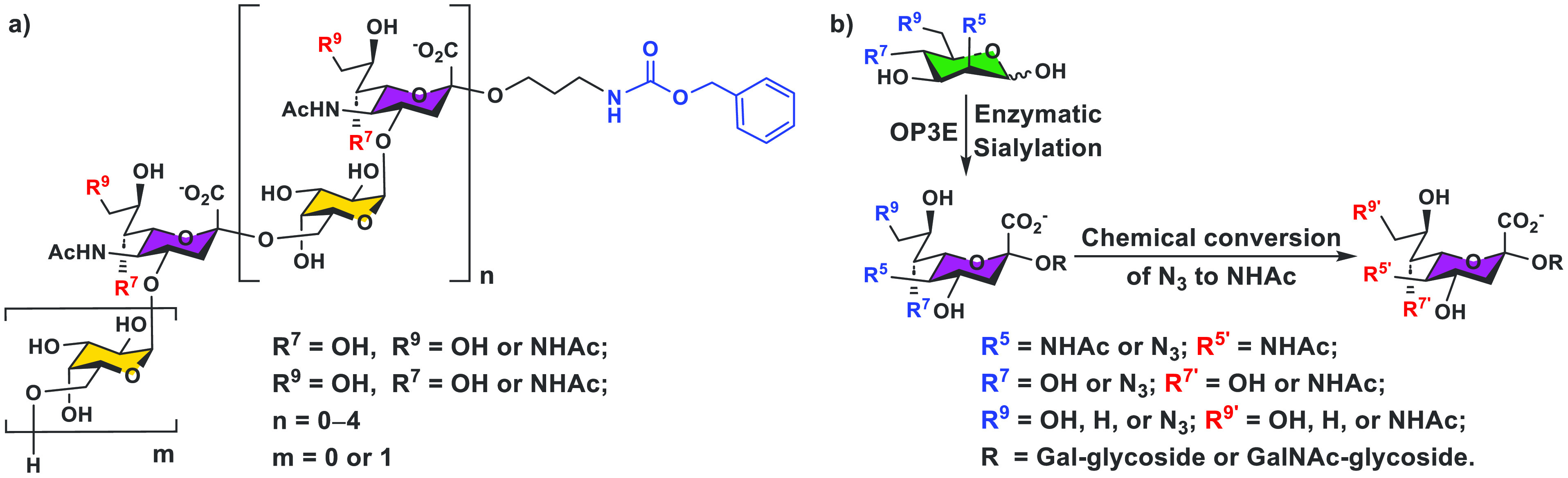
a) Chemoenzymatically synthesized NHCbz-tagged oligosaccharides of *Neisseria meningitidis* serogroup W CPS with a disaccharide repeat -6Galα1–4Siaα2-, including those containing the *N*-acetyl analogs of 9-*O*- and/or 7-*O*-acetylated Neu5Ac and b) chemoenzymatic synthon strategy for producing NHAc-containing sialosides from an N_3_-containing monosaccharide by a OP3E enzymatic sialylation reaction followed by the chemical conversion of N_3_ to NHAc.

Engineering glycosyltransferase acceptor substrate with a Cbz tag^29^ also worked well for synthesizing glycans with an alkylamine aglycone that can be used for printing glycan microarrays^46^ or for conjugation with proteins and other molecules. Such a strategy was applied effectively for the chemoenzymatic total synthesis and purification of oligosaccharides (varying from disaccharide to decasaccharide) of sialic acid-containing CPS of *Neisseria meningitidis* serogroup W (Figure 8a).^47^ Azido group-modified ManNAc and mannose derivatives were effective chemoenzymatic synthons for synthesizing NHAc-containing nonulosonic acids (e.g., Leg5,7diNAc)^26^ and derivatives (e.g., Neu5Ac9NAc, Neu5Ac7NAc, and Neu5Ac7,9diNAc, which are stable analogs of the corresponding *O*-acetylated Neu5Ac) and the corresponding glycosides (Figure 8b).^41,46,48^ The combined chemoenzymatic synthon and glycosyltransferase acceptor engineering strategies were applied successfully for synthesizing stable *N*-acetyl analogs of *Neisseria meningitidis* serogroup W CPS oligosaccharides containing 9-*O*- and/or 7-*O*-acetylated Neu5Ac.^48^

## Biocatalyst Identification and Engineering

Successful enzymatic and chemoenzymatic synthesis of complex sialylated glycans and glycoconjugates in sufficient amounts relies on access to enzymes (especially sialyltransferases) and their engineered mutants with desired properties in large amounts inexpensively. We chose *E. coli* as the host to express sialoside biosynthetic enzymes due to its ease of handling, low cost, and short time frame for enzyme production. Based on their amino acid sequence similarities, sialyltransferases are mainly grouped into glycosyltransferase GT families 29, 38, 42, 52, 80, 97, and 100 in the carbohydrate active enzyme database (CAZy, www.cazy.org). As glycosyltransferases from bacterial sources are generally more adaptable for *E. coli* expression, we initially focused on identifying, expressing in *E. coli*, biochemically and structurally characterizing, and engineering bacterial sialyltransferases. General strategies that we applied and worked well in expressing soluble and active enzymes in *E. coli* include cloning using synthetic genes with codons optimized for *E. coli* expression as templates for polymerase chain reactions, exploring the location of the His_6_-tag at N- or C-terminus, considering amino acid truncations at the N- and/or C-terminus, adding a maltose binding protein (MBP) fusion component at the N-terminus, varying the concentration of inducers, lowering the expression temperature after induction, expressing enzymes in BL21(DE3) or Origami B(DE3) cells with or without the coexpression of chaperones, adding betaine to the expression medium, or using 2YT instead of LB medium. More recently, we also succeeded in expressing mammalian sialyltransferases including those from humans^44,49^ in *E. coli* with N-terminal amino acid truncation and fusion with an N-terminal MBP. The coexpression of chaperones in Origami B (DE3) cells was also helpful.^44^ Enzymes that we have studied and used for OPME sialylation are summarized in Table 1.

**Table 1:** Enzyme and Mutants Expressed in *E. coli* and Used for the Synthesis of Sialosides in the Chen Laboratory

**Abbreviated name with ref, CAZy family, crystal structure ref**	**Source**	**Synthetically useful mutants with ref**
**Sialyltransferases and mutants for synthesizingα2–3-linkedsialosides**		
PmST1^1^ (GT80)	*Pasteurella multocida*	PmST1_M144D (with crystal structure)^2^
Crystal structures^51,52^		PmST1_E271F/R313Y^53^
PmST2^56^ (GT52)	*Pasteurella multocida*	
PmST3^57^ (GT42)	*Pasteurella multocida*	
Hd2,3ST (Hd0053)^49^ (GT80)	*Haemophilus ducreyi*	
vST3Gal-I^59^ (GT29)	myxoma virus-infected European rabbit kidney RK13 cells	
hST3GAL-II^42^ (GT29)	human	
CjCst-I^41,44^ (GT42)	*Campylobacter jejuni*	
**Sialyltransferases and mutants for synthesizingα2–6-linkedsialosides**		
Pd2,6ST^11,60,61^ (GT80)	*Photobacterium damselae*	Pd2,6ST_A200Y/S232Y^66^
Crystal structures^67^		Pd2,6ST_S232L/T356S/W361F as an α2–6-neosialidase^62^
Psp2,6ST^63^ (GT80)	*Photobacterium* species	Psp2,6ST_A366G^68^
hST6GAL-I^44^ (GT29)	human	
NmSiaD_w_^47^ (GT97)	*Neisseria meningitidis*	
PmST1_P34H/M144 V^65^ (GT80)	*Pasteurella multocida*	
**Sialyltransferases for synthesizingα2–8-linkedsialosides**		
CjCst-II^69^ (GT42)	*Campylobacter jejuni*	
**Sialic acid aldolases**		
EcAldolase^70^	*Escherichia coli*	
PmAldolase^71^	*Pasteurella multocida*	
Crystal structures^72^		
**CMP-Nonulosonic acid synthetases**		
NmCSS^70^	*Neisseria meningitidis*	NmCSS_S81R and NmCSS_Q163A^73^
Crystal structures^74^		
EcCSS^70^	*Escherichia coli*	
SaVCSS^70^	*Streptococcus agalactiae*	
PmCSS^73^	*Pasteurella multocida*	
HdCSS^73^	*Haemophilus ducreyi*	
LpCLS^75^	*Legionella pneumophila*	

The first sialyltransferase that we cloned and characterized was a multifunctional α2–3-sialyltransferase from *Pasteurella multocida* (PmST1). PmST1 (60 U/mg) is the most active sialyltransferase that has been characterized to date. It has promiscuity toward both donor and acceptor substrates. Its main function is an α2–3-sialyltransferase, but its other functionalities,^1^ including weaker α2–6-sialyltransferase activity, donor hydrolysis, and sialidase and trans-sialidase activities contributed mainly by its reverse sialylation activity^50^ can complicate the synthetic process. Applying PmST1 in synthesis requires controlling the amount of enzyme used, reaction time, pH, temperature, and other conditions to achieve high yields. Crystal structure studies by collaboration with Andrew Fisher^51,52^ and mutagenesis studies led to the designing of the PmST1_E271F/R313Y double mutant^53^ and PmST1_M144D single mutant^2^ with decreased sialidase and/or donor hydrolysis activities, thus improving their application in synthesis. PmST1_M144D is especially useful for directly α2–3-sialylating fucosylated galactosides for the synthesis of sialyl Lewis x^2^ and sialyl Lewis a^54^ glycans with^55^ or without additional *O*-sulfation on the Gal or GlcNAc. Unlike PmST1 and mutants which do not use lactosyl sphingosine and derivatives efficiently as acceptor substrates, the second α2–3-sialyltransferase from *Pasteurella multocida* (PmST2)^56^ prefers glycolipids but not glycans as acceptors. On the other hand, the third *Pasteurella multocida* α2–3-sialyltransferase (PmST3)^39,43,57^ and *Campylobacter jejuni* α2–3-sialyltransferase CjCst-I^41,44^ use glycans, glycolipids, and glycopeptides/glycoproteins as acceptors efficiently. While PmST1, PmST1_M144D, and CjCst-I can use both β1–4- and β1–3-linked galactosides as acceptor substrates efficiently, PmST3 has been shown to selectively α2–3-sialylate the β1–4-linked galactoside branch, but not the β1–3-linked galactoside branch, on a Core 2 glycan,^58^ although it was capable of α2–3-sialylating the unbranched Core 1 T antigen (a β1–3-linked galactoside) on glycopeptides.^43^ Human ST3GAL-II uses β1–3-linked galactosides as acceptor substrates efficiently for the high-yield synthesis of the glycans of gangliosides GT1b, GD1a, and GM1b.^42^ With lower expression levels, recombinant *Haemophilus ducreyi* α2–3-sialyltransferase Hd2,3ST^49^ and a recombinant viral α2–3-sialyltransferase vST3Gal-I^59^ have been used less frequently for synthesis.

*Photobacterium damselae* α2–6-sialyltransferase (Pd2,6ST)^11,60,61^ is another broadly used sialyltransferase for synthesis. It also has sialidase and trans-sialidase activities mainly contributed by its reverse sialylation activity,^50^ and the reaction progress needs to be monitored and stopped promptly. In fact, its α2–6-sialidase activity was enhanced by site-specific saturation mutagenesis to produce Pd2,6ST_S232L/T356S/W361F mutant as an α2–6-neosialidase^62^ which can facilitate sialoglycan analysis. *Photobacterium* species α2–6-sialyltransferase (Psp2,6ST)^63^ was found to be more effective than Pd2,6ST in using α-linked *N*-acetylgalactosaminides such as Tn antigens as acceptor substrates. Both Pd2,6ST and Psp2,6ST are promiscuous toward donor substrate modifications and can add sialic acids α2–6-linked to both terminal and internal β-linked Gal and/or GalNAc residues.^64^ This property has been used as an enzymatic protection group strategy to protect internal Glc or GlcNAc in long-chain galactosides from enzymatic fucosylation.^10^ To selectively add sialic acid α2–6-linked to only the terminal Gal or GalNAc, we developed the PmST1_P34H/M144 V^65^ double mutant and the groups of Jiansong Cheng and Hongzhi Cao developed Pd2,6ST_A200Y/S232Y.^66^ The latter with a higher regiospecificity is especially useful for synthetic purposes.^3^ Based on the crystal structures of Pd2,6ST^67^ and structural modeling, the Psp2,6ST_A366G mutant^68^ with a better expression level was designed and confirmed as an improved catalyst for synthesis. For the formation of the α2–6-sialyl linkage in disialylated biantennary complex-type *N*-glycans on glycoproteins, recombinant human α2–6-sialyltransferase hST6GAL-I expressed in *E. coli* was found to be efficient.^44^ Bifunctional glycosyltransferase NmSiaD_w_ from *Neisseria meningitidis* serogroup W was successfully used for the synthesis of its CPS oligosaccharides^47^ containing α2–6-linked Neu5Ac or its derivative with an *N*-acetyl group at C7 or C9 as stable mimics of those containing labile Neu5Ac *O*-acetyl groups.^48^

Bifunctional *Campylobacter jejuni* α2–3/8-sialyltransferase CjCst-II^69^ has been broadly used for its α2–8-sialyltransferase activity for the synthesis of α2–8-sialosides including gangliosides^39,41^ and glycans.^18,35^

The application of sialyltransferases in synthesis requires the use of the corresponding sugar nucleotide CMP-Sia. Those containing a sialic acid form other than Neu5Ac are not commercially available. Therefore, access to highly efficient CMP-Sia biosynthetic enzymes is critical to the chemoenzymatic synthesis of sialosides, especially those containing a sialic acid or derivative other than Neu5Ac. Bacterial sialic acid aldolases and CMP-sialic acid synthetases (CSSs) work well for synthetic purposes. Both EcAldolase^70^ and PmAldolase^71,72^ were efficiently for the formation of a diverse arrays of sialic acids and analogs from their six-carbon precursors, and the latter was shown to be more effective for some substrates.^33,71^

Among five bacterial CSSs^70,73^ that we characterized, NmCSS^74^ has the highest catalytic efficiency^73^ and its expression level (100 mg/L culture) is comparable to that of PmCSS (90 mg/L culture) or HdCSS (110 mL/culture).^73^ Its substrate promiscuity and catalytic efficiency have been further improved by developing NmCSS_S81R and NmCSS_Q163A mutants.^73^ For the synthesis of CMP-Leg5,7Ac_2_ from CTP and Leg5,7Ac_2_ prepared from 6deoxyManNAc4NAc, NmCSS is not efficient and a *Legionella pneumophila* CMP-legionaminic acid synthetase^75^ has been used effectively with PmAldolase and PmST1 for the synthesis of Leg5,7diNAc-containing glycosides.

## Conclusions and Perspectives

One-pot multienzyme (OPME) systems with the combination of sialyltransferase donor and acceptor substrate engineering, biocatalyst identification and engineering, and process engineering strategies are enabling approaches to access structurally complex, biologically important sialosides containing different sialic acid forms, various sialyl linkages, and diverse underlying glycans and glycoconjugates. The availability of structurally defined sialosides has allowed and will continue to empower the investigation of their multifaceted functions at the molecular level and the exploration of their applications. Continuous efforts in chemoenzymatic method development including process automation as well as identifying, characterizing, engineering, and designing sialyltransferases, other glycosyltransferases, and related sugar nucleotide biosynthetic enzymes with desired properties (such as high activity, stability during storage and in reactions, feasibility to be produced in large amounts inexpensively, and tolerance for substrate modifications while retaining regio- and stereospecificities for the formation of desired glycosidic linkages) remain to be critical for the successful chemoenzymatic synthesis of carbohydrates including those found in eukaryotes, bacteria, and other organisms to drive glycoscience and the related fields forward.
